# Changes in the food environment over time: examining 40 years of data in the Framingham Heart Study

**DOI:** 10.1186/s12966-017-0537-4

**Published:** 2017-06-24

**Authors:** Peter James, Michael W. Seward, A. James O’Malley, SV Subramanian, Jason P. Block

**Affiliations:** 1 Division of Chronic Disease Research Across the Life Course (CoRAL), Department of Population Medicine, Harvard Medical School and Harvard Pilgrim Health Care Institute, 401 Park Dr Suite 401, Boston, MA 02215 USA; 2 Department of Biomedical Data Science, The Dartmouth Institute, Geisel School of Medicine at Dartmouth, Lebanon, NH USA; 3 Department of Social and Behavioral Sciences, Harvard TH Chan School of Public Health, Boston, MA USA; 4 Harvard Center for Population and Development Studies, Harvard University, Boston, MA USA

**Keywords:** Food environment, Fast food, Supermarkets, Geographic information systems, Prospective cohort study

## Abstract

**Background:**

Research has explored associations between diet, body weight, and the food environment; however, few studies have examined historical trends in food environments.

**Methods:**

In the Framingham Heart Study Offspring (*N* = 3321) and Omni (*N* = 447) cohorts, we created food environment metrics in four Massachusetts towns utilizing geocoded residential, workplace, and food establishment addresses from 1971 to 2008. We created multilevel models adjusted for age, sex, education, and census tract poverty to examine trends in home, workplace, and commuting food environments.

**Results:**

Proximity to and density of supermarkets, fast-food, full service restaurants, convenience stores, and bakeries increased over time for residential, workplace, and commuting environments; exposure to grocery stores decreased. The greatest increase in access was for supermarkets, with residential distance to the closest supermarket 1406 m closer (95% CI 1303 m, 1508 m) by 2005–2008 than in 1971–1975. Although poorer census tracts had higher access to fast-food restaurants consistently across follow-up, this disparity dissipated over time, due to larger increases in proximity to fast-food in wealthier neighborhoods.

**Conclusions:**

Access to most food establishment types increased over time, with similar trends across home, workplace, and commuter environments.

**Electronic supplementary material:**

The online version of this article (doi:10.1186/s12966-017-0537-4) contains supplementary material, which is available to authorized users.

## Background

The prevalence of obesity in the United States has risen rapidly over past decades, and research links this rise to social and environmental factors [1–3]. Studies have attempted to estimate how neighborhood food environments might drive changes in diet and body mass index (BMI) [4]. Changes to the food environment may be related to increases in away-from-home food expenditures [5], consumption of fast-food and sugar-sweetened beverages [6], and larger portion sizes [7]; however, few longitudinal studies have examined trends in food environments over the past 40 years.

Recent studies have found that supermarket availability is negatively associated with obesity while fast-food availability is linked to higher BMI [4]. Despite a large number of studies, evidence for associations between food environments and obesity is still inconsistent [4, 8–10]. Food environment studies typically have several shortcomings, including reliance on inaccurate commercial databases for food establishment data, heterogeneity of food environment metrics (e.g., proximity versus density), and a reliance on cross-sectional study designs. Additionally, these studies generally focus on the food environment around the home, disregarding other locations that might be relevant to health, including the work and commuting environment [4]. Understanding changes in the food environment over time, as well as using multiple metrics of exposure, would provide new perspectives to estimate how access to food influences health.

In this study, we analyzed almost 40 years of historical information (1971–2008) on trends in food environments including six types of establishments: fast-food restaurants, full service restaurants, bakeries and coffee shops, chain supermarkets, independent grocery stores and farmer’s markets, and convenience stores. In four Massachusetts towns, we evaluated proximity as well as density of food establishments, and created metrics based on the home, workplace, and likely commuting route from work to home. In addition, we evaluated age, poverty, sex, and education to determine whether these factors were related to disparities in food environment exposures over time.

## Methods

### Population

This study used data from the Framingham Heart Study (FHS) Offspring Cohort and the first Omni Cohort. The FHS Offspring Cohort began in 1971 with 5124 subjects who were either the children of subjects enrolled in the FHS Original Cohort or their spouses [11]. The FHS Original Cohort enrolled a random sample of residents of Framingham, Massachusetts in the 1940s [12]. Offspring Cohort subjects have been examined and surveyed up to eight times from enrollment through 2008, roughly every four years. Since the FHS Offspring Cohort began, the community of Framingham has evolved. In the early 1990s, the need to establish a new group of participants reflecting the increasing diversity of the community was recognized. The first Omni Cohort started in 1994, and included 507 men and women of African-American, Hispanic, Asian, Indian, Pacific Islander, and Native American race/ethnicity living in and around Framingham [13]. The Omni Cohort was sampled in concert with the Offspring Cohort through 2008.

Our final sample included Offspring and Omni Cohort subjects, excluding observations with missing census tract of residence (primarily in the 1970s when some areas had not yet been characterized into tracts), or when a participant was lost to follow-up. For the home food environment analysis (Additional file 1: Figure S1), we further restricted the sample to 15, 373 observations (3567 participants) in which a participant lived in the four towns with the most FHS participants: Framingham, Ashland, Holliston, and Natick, Massachusetts. We focused on these towns to allow for validation of food establishment data. For the workplace analysis, we restricted the sample to 7357 observations (2447 participants) where the participant worked in these four towns. For the analysis of likely commuter routes from work to home, we restricted the sample to those who lived and worked in the four town area (6481 observations; 2187 participants).

### Food environment

We evaluated six food establishment types: fast-food restaurants, full service restaurants, bakeries and coffee shops, chain supermarkets, independent grocery stores and farmer’s markets, and convenience stores. We defined these establishment types in accordance with the North American Industry Classification System [14]. Additional file 1: Table S1 shows the NAICS codes that were used as guidelines to categorize food establishments. We explored changes in the food environment over time from 1) home; 2) workplace; and 3) during the commute from work to home for each establishment type.

We collected food establishment names, addresses, type, and years of operation from multiple sources: files of open and closed food establishments maintained by the four towns’ local boards of health, which conduct food safety inspections; historical Framingham-area Yellow Pages; historical Framingham-area White Pages; and a commercial database compiled by Dun & Bradstreet (Short Hills, New Jersey) for selected years from each historical wave [15]. We collected and geocoded all data for the four-town area, as well as for the 10 additional towns that surround the area (which could be a source of food establishment exposure for subjects living near the borders of these towns). Using board of health data as our gold standard, we validated the final food establishment database through site visits in 2008 and 2012–2013 to establishments that were open at the time and by review of local boards of health and Framingham Study staff. More detail on our methodology can be found in a previous publication [15].

We gathered participant home and workplace addresses from FHS records. For participants who provided a workplace name but not an address, we examined historical yellow pages and internet sources to identify addresses. With this geocoded information and food establishment data, we created multiple food environment measures: driving distance, buffer density, and commuting exposure using ArcGIS, version 9.3 (Esri, Redlands, California). For each food establishment type, we estimated the driving distance via the road network from the home or workplace to the closest establishment. As a measure of food establishment density, we counted each type of establishment within a 1500 m radial buffer of home and workplace addresses. For commuting exposure, we counted establishments within a 60 m buffer along the fastest driving route (determined by distance and speed limit) between work and home.

### Cov*a*riates

Individual-level covariates were included in models a priori: age (years), sex, and education (less than high school, greater than high school, or missing) based on exam data. We included a category for missing education because this measure was only captured during waves 2, 3, and 8 for the Offspring cohort; many Offspring participants did not have this information because they did not attend an exam during those waves or did not provide information. We accounted for area-level socioeconomic status by including the percent of census tract residents below the federal poverty line, based on the geocoded home or workplace addresses [16]. This measure of census tract poverty was time-varying, and we fixed census tract borders to the 2000 borders to ensure geographic stability over time.

### Statistical analysis

Results are presented focusing on fast-food and supermarket establishments, as these establishments have been highlighted in the literature; however, results on all establishments are included in supplemental tables. First, we created plots of each food environment metric for each establishment type over follow-up. We then created cross-classified multilevel models with indicator variables for each wave of follow-up, adjusted for year of birth, sex, education, and time-varying census tract poverty. Multilevel models accounted for clustering of observations within individuals and census tracts over time; within individual clustering was captured with a random slope for linear time. We entered levels as *cross-classified* to account for moving, such that an individual could live or work in a different census tract at each wave of follow-up. Additional models tested interactions between time and all covariates. For a visual aid, we created figures with predicted estimates for a female participant of mean age with greater than a high school education, and stratified estimates by levels of census tract poverty (one standard deviation below the mean, mean, and one standard deviation above the mean).

We analyzed models using Markov chain Monte Carlo methods in MLWin, version 2.34 (Centre for Multilevel Modelling, University of Bristol, United Kingdom). These models generated multiple iterative samples from the joint posterior distribution of the parameters. Resulting output from models included parameter estimates and 95% credible intervals that reflected the joint posterior distribution of the parameter estimates. We defined *significant* associations as those predictors or interaction terms with estimated parameters whose 2-sided 95% credible intervals did not include 0. We generated descriptive results using SAS, version 9.3 (Cary, North Carolina). The institutional review board of Harvard Medical School approved this study. Data were analyzed in 2016.

## Results

Across all analyses, this study included 3321 participants from the FHS Offspring cohort and 447 Omni participants (Additional file 1: Figure S1). The mean number of observations per subject was 4.3 for participants in the home analysis, and 3.0 for participants in the workplace analysis, and 3.0 for participants in the commute analysis. The mean age of Offspring participants at enrollment was 38 years; the mean age of Omni participants at enrollment was 52 years (Table 1). Approximately 50% of Offspring participants were female, while 58% of Omni participants were female.

**Table 1:** Participant characteristics

	Offspring cohort (*N* = 3321)	Omni cohort (*N* = 447)
	Mean (SD)	Mean (SD)
Age (years)	38.4 (10.4)	51.6 (9.3)
Home Census Tract Percent Below Poverty	6.4 (4.4)	8.0 (5.0)
Workplace Census Tract Percent Below Poverty	6.7 (4.3)	7.4 (4.8)
	*N* (%)	*N* (%)
Education		
< High School	1476 (44.4)	33 (7.4)
> High School	1454 (43.8)	218 (48.8)
Missing	391 (11.8)	196 (43.9)
Sex		
Male	1647 (49.6)	186 (41.6)
Female	1674 (50.4)	261 (58.4)

### Home food environment

**Fig. 1:**
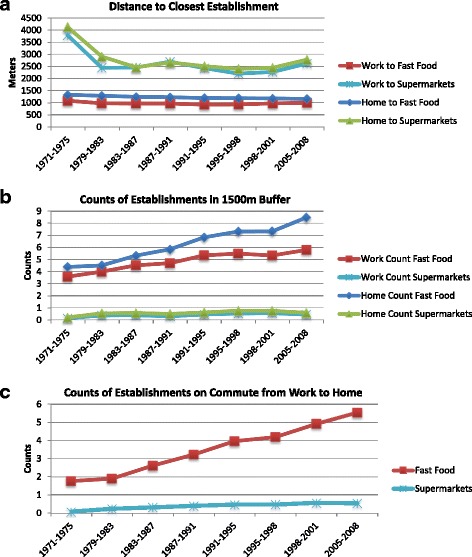
Access to fast-food and supermarkets over time for (**a**) distance to the closest establishment (**b**) counts of establishments within a 1500m buffer, and (**c**) counts of establishments within a 60m buffer of the commute between work and home

In the 1970s, participants lived closer to fast-food restaurants, full service restaurants, and convenience stores than grocery stores, bakeries, and supermarkets (Fig. 1a, Additional file 1: Figure S2). Over time, the distance to most food establishment types decreased, except for grocery stores. The mean residential distance to the nearest supermarket declined from 4125 m (SD 2650 m) in 1971–75 to 2774 m (SD 1422 m) in 2005–08, while the mean distance to grocery stores increased from 1577 m (SD 1224 m) to 2179 m (SD 1344 m). Density measures mirrored those of proximity measures; in 1971–75, counts of full service restaurants in the 1500 m buffer around the home were highest (7.1 restaurants per 1500 m buffer), followed by fast-food, grocery stores, convenience stores, bakeries and coffee shops, and then supermarkets (Fig. 1b, Additional file 1: Figure S3). Over time, the density of convenience stores, fast-food, full service restaurants, and bakeries increased and supermarket density remained relatively constant, while the density of grocery stores decreased from 3.4 stores (SD 3.4) per 1500 m buffer in 1971–75 to 1.8 stores (SD 2.2) in 2005–08.

Cross-classified multilevel models indicated that sex and age were not related to home food access, while higher education was associated with lower access to all food types (results not shown). In models adjusted for age, sex, education, and census tract poverty, we observed increases in proximity to and density of fast-food, full service restaurants, convenience stores, and bakeries over time (Table 2, Additional file 1: Table S2). In 1971–1975, the adjusted mean distance from home to the closest establishment was 1406 m [95% CI 1250, 1562] for fast-food and 4547 m [95% CI 4154, 4939] for supermarkets. By 2005–08, distance from home to the closest establishment declined by 298 m [95% CI −336, −259] for fast-food and 1406 m [95% CI −1509, −1302] for supermarkets. Distance to the nearest grocery store in 1971–75 was 1656 m [95% CI 1340, 1972], and increased over time by 342 m [95% CI 278, 406] in 2005–08. Results were consistent for density measures.

**Table 2:** Main results for multilevel models examining food environments over time

	Fast-food				
	Home		Work		Commute
	Distance from Closest	Counts within 1500 m Buffer	Distance from Closest	Counts within 1500 m Buffer	Counts within 60 m Buffer
	Change in Meters (95% CI)	Change in Count (95% CI)	Change in Meters (95% CI)	Change in Count (95% CI)	Change in Count (95% CI)
Wave 1 (1971–1975)	Ref	Ref	Ref	Ref	Ref
Wave 2 (1979–1983)	−62.3 (−80.1, −44.4)	0.4 (0.3, 0.5)	−49.9 (−73.2, −26.6)	0.5 (0.3, 0.6)	0.0 (−0.1, 0.2)
Wave 3 (1983–1987)	−141.4 (−163.5, −119.3)	1.3 (1.2, 1.5)	−106.1 (−131.3, −80.9)	1.4 (1.2, 1.5)	0.7 (0.5, 0.9)
Wave 4 (1987–1991)	−168.0 (−191.1, −144.9)	2.0 (1.9, 2.1)	−139.3 (−168.4, −110.2)	1.8 (1.6, 2.0)	1.1 (0.9, 1.3)
Wave 5 (1991–1995)	−215.8 (−241.7, −190.0)	3.1 (2.9, 3.2)	−202.5 (−237.2, −167.8)	3.1 (2.8, 3.3)	1.6 (1.4, 1.9)
Wave 6 (1995–1998)	−213.5 (−242.1, −184.8)	3.3 (3.2, 3.5)	−200.7 (−238.3, −163.0)	3.3 (3.1, 3.6)	1.7 (1.4, 2.0)
Wave 7 (1998–2001)	−262.1 (−294.4, −229.7)	3.7 (3.5, 3.9)	−225.6 (−271.4, −179.8)	3.6 (3.3, 3.9)	2.0 (1.6, 2.3)
Wave 8 (2005–2008)	−297.8 (−336.4, −259.2)	4.9 (4.7, 5.1)	−276.9 (−334.8, −219.0)	4.5 (4.1, 4.9)	2.4 (1.9, 2.9)
	Supermarkets				
	Home		Work		Commute
	Distance from Closest	Counts within 1500 m Buffer	Distance from Closest	Counts within 1500 m Buffer	Counts within 60 m Buffer
	Change in Meters (95% CI)	Change in Count (95% CI)	Change in Meters (95% CI)	Change in Count (95% CI)	Change in Count (95% CI)
Wave 1 (1971–1975)	Ref	Ref	Ref	Ref	Ref
Wave 2 (1979–1983)	−1193.7 (−1238.2, −1149.2)	0.3 (0.3, 0.3)	−1432.2 (−1491.8, −1372.6)	0.2 (0.2, 0.3)	0.1 (0.1, 0.1)
Wave 3 (1983–1987)	−1680.3 (−1735.7, −1624.9)	0.3 (0.3, 0.4)	−1637.2 (−1699.4, −1575.0)	0.3 (0.2, 0.3)	0.2 (0.1, 0.2)
Wave 4 (1987–1991)	−1523.4 (−1582.2, −1464.6)	0.3 (0.2, 0.3)	−1142.2 (−1212.7, −1071.8)	0.1 (0.1, 0.2)	0.3 (0.2, 0.3)
Wave 5 (1991–1995)	−1680.4 (−1747.5, −1613.4)	0.4 (0.4, 0.4)	−1728.3 (−1813.3, −1643.4)	0.4 (0.3, 0.4)	0.3 (0.3, 0.4)
Wave 6 (1995–1998)	−1711.1 (−1785.3, −1637.0)	0.6 (0.5, 0.6)	−1724.0 (−1811.5, −1636.5)	0.5 (0.4, 0.5)	0.3 (0.3, 0.4)
Wave 7 (1998–2001)	−1720.9 (−1806.8, −1635.0)	0.6 (0.5, 0.6)	−1740.0 (−1850.2, −1629.8)	0.5 (0.4, 0.5)	0.3 (0.3, 0.4)
Wave 8 (2005–2008)	−1405.7 (−1508.7, −1302.7)	0.4 (0.3, 0.4)	−1401.5 (−1540.8, −1262.3)	0.3 (0.3, 0.4)	0.3 (0.2, 0.4)

Note: All analyses are adjusted for age, sex, education, and census tract poverty

### Workplace food environment

We observed similar trends around the workplace food environment (Fig. 1a and b, Additional file 1: Figures S4–S5). Distance to the nearest supermarket decreased from 3606 m (SD 2027 m) in 1971–75 to 2302 m (SD 1173 m) in 2005–08, while distance to the closest grocery store increased from 999 m (SD 905 m) to 1495 m (SD 1220 m). Distance to the closest full service restaurant increased slightly from 617 m (SD 573 m) in 1971–75 to 668 m (SD 612 m) in 2005–08. Distance to the closest fast-food, convenience store, and bakery decreased, although as with residential addresses, the degree of decline was much less than with supermarkets.

Multilevel models for workplace food environments were consistent with patterns observed for home-based analyses. Participant age and sex were not strongly linked to workplace food access, while higher education was consistently linked to lower workplace access to food establishments (results not shown). In 1971–75, the adjusted mean distance from the workplace to the closest establishment was 987 m [95% CI 816, 1158] for fast-food and 4445 m [95% CI 4003, 4887] for supermarkets. By 2005–08, workplace distance to the closest establishment declined by 277 m [95% CI −335, −219] for fast-food, and 1402 m [95% CI −1541, −1262] for supermarkets (Table 2). Distance from the workplace to the nearest grocery store in 1971–1975 was 1302 m [95% CI 979, 1626], and increased over time by 258 m [95% CI 162, 354] (Additional file 1: Table S3). Findings for density measures were consistent with those for proximity measures.

### Commute food environment

Trends in access along the commute from work to home were similar to those in the home and workplace analyses (Fig. 1c, Additional file 1: Figure S6). The number of convenience stores, fast-food, and full service restaurants along the commute steadily increased over time with little change for bakeries, supermarkets, and grocery stores. In multilevel models (Table 2, Additional file 1: Table S4), in 1971–75, the mean number of establishments passed on community routes from work to home was 1.7 [95% CI 1.1, 2.2] for fast-food, 0.1 [95% CI 0.0, 0.1] for supermarkets, and 1.3 [95% CI 0.8, 1.8] for grocery stores. By 2008, commute density increased by 2.4 [95% CI 1.9, 2.9] for fast-food and 0.3 [95% CI 0.2, 0.4] for supermarkets. Commute density decreased by 0.4 [95% CI −0.6, −0.1] for grocery stores over follow-up.

### Effect modification

**Fig. 2:**
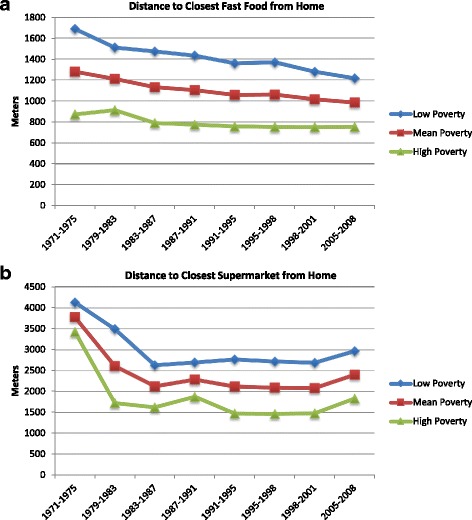
Predicted value of mean distance from home to **a** closest fast-food and **b** closest supermarket stratified by Census tract poverty. Note: Poverty categories: 1SD below mean poverty (2.2%), Mean poverty (6.6%), 1SD above mean poverty (11.0%)

There was no consistent evidence that trends in food establishment proximity or density around the home varied by sex, age, or education (results not shown). However, we observed consistent differences in proximity to food establishments over time by levels of census tract poverty. Analyses stratified by census tract poverty (poverty one SD below the mean (2.2%), mean poverty (6.6%), and poverty one SD above the mean (11.0%)) showed that home proximity to fast-food increased over follow-up across all levels of poverty (Fig. 2a). While home proximity to fast-food was highest for participants living in the poorest census tracts at all time periods, proximity to fast-food increased more rapidly for low poverty census tracts such that disparities in proximity to fast-food were smaller by 2005–2008. Home proximity to supermarkets was consistently highest across all time periods for those living in the poorest census tracts (Fig. 2b), although proximity to supermarkets across all levels of poverty increased markedly from 1971 to 1975 to 1983–87 and then plateaued. Disparities in home proximity to supermarkets by levels of poverty increased slightly by the end of follow-up. Findings were similar for density measures. Similarly, workplaces in poorer census tracts had higher proximity to fast-food and lower proximity to supermarkets at baseline and this disparity vanished over follow-up (Additional file 1: Figure S7). There was little evidence that trends in food establishment proximity or density around the workplace varied by sex, age, or education (results not shown).

## Discussion

In this analysis of 37 years of validated food environment data, we observed increases over time in proximity to and density of supermarkets, fast-food, full service restaurants, bakeries, and convenience stores. Proximity to smaller grocery stores decreased over time. On average, food establishments were closer but had lower densities around workplace addresses compared to home addresses at all time points over follow-up. This finding is contrary to previous analyses that showed substantially higher density of food establishments around the workplace [17]. For the density of food establishments on commuting routes, trends over time paralleled those observed for density of food establishments around the home and workplace. Although those living in the poorest census tracts had greater access to fast-food over follow-up, access to fast-food increased more rapidly for those living in low poverty census tracts such that disparities in proximity to fast-food diminished over time. Workplaces in poorer census tracts had lower proximity to supermarkets and higher proximity to fast-food at baseline, but this disparity disappeared over time.

Few studies have evaluated historical trends in the food environment. Gibson used data from the National Longitudinal Survey of Youth from 1998 to 2004 and created zip code-level density measures of supermarkets, grocery stores, convenience and specialty food stores, limited-service restaurants, and full-service restaurants from US Census Data [18]. Overall, the authors observed small decreases in density across all food metrics. Richardson et al. examined food environments in the US-based Coronary Artery Risk Development in Young Adults study from 1985 to 2006, and found that, consistent with our findings, the availability of fast food and non-fast food restaurants and supermarkets and convenience stores increased over follow-up [19]. However, contrary to our results, the authors observed that lower SES neighborhood residents had fewer fast food and non-fast food restaurants, more convenience stores, and the same number of supermarkets in their neighborhoods compared to wealthier neighborhoods. A previous analysis of Framingham Offspring cohort data through 2001 found that the mean driving distance from the home address to fast-food, full service restaurants, and supermarkets decreased over time, while driving distances to grocery stores slightly increased [15]. These findings are consistent with the current analysis, which is extended through 2008 and includes the Omni Cohort.

The findings from this study may inform the conversation over the primacy of food deserts, areas lacking access to nutritious and affordable food, versus food swamps, areas where large relative amounts of energy-dense snack foods inundate healthy food options [20–22]. We observed that participants from the poorest census tracts had higher proximity to fast-food at all time periods, and that access to supermarkets was highest for the poorest census tracts. In addition, access to fast food increased dramatically for all participants over follow up. These findings suggest that, in this context, food swamps are the predominant concern.

### Limitations

This study had several limitations. Due to the extensive effort to identify historical food establishments, the study area included only four major towns of the Framingham Heart Study. We were not able to follow subjects if they moved out of this area, although data were included if participants moved back into the four town area. Therefore, findings may reflect phenomena specific to the four town area, and may not be generalizable to all communities in which FHS participants lived or worked. The lack of racial diversity in the Offspring Cohort is a limitation; however, the Omni Cohort included an ethnically diverse sample and enhances the generalizability of our findings. In analyses of the workplace food environment, adjustment for the poverty levels of census tract residents may not fully capture the area-level economic status of a workplace. Therefore, residual confounding by socioeconomic factors may still exist for these analyses. In addition, we were unable to validate historical food data prior to 2008. Our analysis was also limited to measures of the food environment; we did not measure where participants actually purchased food. Future longitudinal studies should query where participants purchase food to investigate how trends in food environments affect actual use of food establishments.

### Strengths

Our study addresses several weaknesses of prior studies. We examined almost 40 years of food environment data. Very few studies on food environments have been longitudinal [4], and those studies were of short duration [18, 23]. Whereas one review found that 87% of studies on food environments and obesity did not validate outlet data in person [4], we incorporated high-quality food environment data from several sources and verified the database through site visits or review by local boards of health and FHS staff. Further, the majority of research to date on food environments has focused on the home or school neighborhood [4]. We examined multiple food environment measures based on both proximity and density of food establishments surrounding the home, workplace, and a novel analysis of the work to home commute. Our analysis of food access based on the commuting and workplace environment offers a more complete picture of the changes in the total food environment over time.

## Conclusions

This analysis of trends in food environment exposures using high quality food establishment data demonstrated that access to most types of food establishments has increased over time, and that although homes in higher poverty neighborhoods had the highest access to fast-food overall, the disparities in fast-food access decreased over time. These findings paint a detailed picture of patterns in access to food, especially when considered in parallel to increases in obesity over the past decades.

## Additional file

Additional file 1: Figure S1. Participant Flow Chart. **Figure S2.** Distance from Closest Establishment from Home. **Figure S3.** Counts of Establishments in 1500 m Buffer around Home. **Figure S4.** Distance from Closest Establishment from Work. **Figure S5.** Counts of Establishments in 1500 m Buffer around Workplace. **Figure S6.** Counts of Establishments in 60 m Buffer around Commute from Workplace to Home. **Figure S7.** Predicted value of mean distance from workplace to a) closest fast-food and b) closest supermarket stratified by workplace Census tract poverty. **Table S1.** NAICS Codes Used to Categorize Food Establishments. **Table S2.** Main Results for Multilevel Models Examining Food Environments over Time based on the Home Address. **Table S3.** Main Results for Multilevel Models Examining Food Environments over Time based on the Workplace Address. **Table S4.** Main Results for Multilevel Models Examining Food Environments over Time based on the Commute from Work to Home (DOCX 50 kb)
