# Supplementary material for: Changes in the food environment over time: examining 40 years of data in the Framingham Heart Study
Source: Int J Behav Nutr Phys Act. 2017 Jun 24;14:84. doi: 10.1186/s12966-017-0537-4 (PMC5483254; doi:10.1186/s12966-017-0537-4)
Supplement: Additional file 1: Figure S1. — Participant Flow Chart. Figure S2. Distance from Closest Establishment from Home. Figure S3. Counts of Establishments in 1500 m Buffer around Home. Figure S4. Distance from Closest Establishment from Work. Figure S5. Counts of Establishments in 1500 m Buffer around Workplace. Figure S6. Counts of Establishments in 60 m Buffer around Commute from Workplace to Home. Figure S7. Predicted value of mean distance from workplace to a) closest fast-food and b) closest supermarket stratified by workplace Census tract poverty. Table S1. NAICS Codes Used to Categorize Food Establishments. Table S2. Main Results for Multilevel Models Examining Food Environments over Time based on the Home Address. Table S3. Main Results for Multilevel Models Examining Food Environments over Time based on the Workplace Address. Table S4. Main Results for Multilevel Models Examining Food Environments over Time based on the Commute from Work to Home (DOCX 50 kb) [file 12966_2017_537_MOESM1_ESM.docx]

Figure S1. Participant Flow Chart

Figure S2. Distance from Closest Establishment from Home

Figure S3. Counts of Establishments in 1500m Buffer around Home

Figure S4. Distance from Closest Establishment from Work

Figure S5. Counts of Establishments in 1500m Buffer around Workplace

Figure S6. Counts of Establishments in 60m Buffer around Commute from Workplace to Home

Figure S7. Predicted value of mean distance from workplace to a) closest fast-food and b) closest supermarket stratified by workplace Census tract poverty

| a |
| --- |
| b |

Note: Poverty categories: 1SD below mean poverty (2.4%), Mean poverty (6.7%), 1SD above mean poverty (11.0%)

Table S1. NAICS Codes Used to Categorize Food Establishments

| Food Establishment | NAICS Code | NAICS Description |
| --- | --- | --- |
| Fast Food Establishments | 722211 | Fast Food  Limited-Service Restaurants |
| Full Service Restaurants | 722110 | Full-Service Restaurants |
| Bakeries and Coffee Shops | 445291 | Baked Goods Stores |
| Supermarkets | 445110 | Supermarkets and Other Grocery (except Convenience) Stores |
| Convenience Stores | 445120 | Convenience Stores |
| Grocery Stores and Farmer’s Markets | 445210 | Meat Markets |
|  | 445220 | Fish and Seafood Markets |
|  | 445230 | Fruit and Vegetable Markets |

Table S2. Main Results for Multilevel Models Examining Food Environments over Time based on the Home Address

| Distance from Closest | | | | |
| --- | --- | --- | --- | --- |
|  | Full Service Restaurant | Bakeries and Coffee Shops | Convenience Store | Grocery Store |
|  | Change in Meters (95% CI) | Change in Meters (95% CI) | Change in Meters (95% CI) | Change in Meters (95% CI) |
| Wave 1 (1971-1975) | Ref | Ref | Ref | Ref |
| Wave 2 (1979-1983) | 0.1 (-15.7, 15.9) | -80.2 (-105.8, -54.5) | -152.1 (-173.0, -131.3) | 148.6 (112.8, 184.4) |
| Wave 3 (1983-1987) | -9.9 (-29.8, 10.0) | -305.5 (-337.3, -273.7) | -261.3 (-286.5, -236.0) | 219.5 (176.4, 262.5) |
| Wave 4 (1987-1991) | -115.9 (-137.3, -94.5) | -316.5 (-349.6, -283.3) | -288.8 (-314.4, -263.2) | 787.8 (744.7, 831.0) |
| Wave 5 (1991-1995) | -132.3 (-156.8, -107.8) | -363.7 (-401.2, -326.3) | -243.3 (-270.9, -215.8) | 496.9 (451.0, 542.8) |
| Wave 6 (1995-1998) | -140.8 (-168.1, -113.5) | -464.7 (-506.2, -423.2) | -209.6 (-239.3, -179.9) | 169.2 (120.9, 217.5) |
| Wave 7 (1998-2001) | -82.9 (-114.4, -51.4) | -481.8 (-529.0, -434.6) | -198.1 (-230.5, -165.6) | 330.2 (277.1, 383.2) |
| Wave 8 (2005-2008) | -68.3 (-105.7, -30.9) | -497.9 (-554.4, -441.4) | -363.4 (-402.7, -324.2) | 341.9 (277.5, 406.2) |
| Counts within 1500m Buffer | | | | |
|  | Full Service Restaurants | Bakeries and Coffee Shops | Convenience Stores | Grocery Stores |
|  | Change in Count | Change in Count | Change in Count | Change in Count |
|  | (95% CI) | (95% CI) | (95% CI) | (95% CI) |
| Wave 1 (1971-1975) | Ref | Ref | Ref | Ref |
| Wave 2 (1979-1983) | 0.6 (0.5, 0.7) | -0.3 (-0.3, -0.2) | 0.4 (0.4, 0.5) | -0.8 (-0.8, -0.7) |
| Wave 3 (1983-1987) | 1.2 (1.1, 1.4) | -0.1 (-0.2, -0.1) | 1.3 (1.2, 1.4) | -0.5 (-0.6, -0.5) |
| Wave 4 (1987-1991) | 2.7 (2.5, 2.9) | 0.0 (-0.1, 0.1) | 1.5 (1.4, 1.6) | -0.7 (-0.8, -0.6) |
| Wave 5 (1991-1995) | 3.8 (3.6, 4.0) | 0.5 (0.4, 0.6) | 2.3 (2.2, 2.5) | -0.6 (-0.7, -0.6) |
| Wave 6 (1995-1998) | 3.9 (3.7, 4.1) | 1.4 (1.3, 1.5) | 3.3 (3.2, 3.5) | -0.9 (-1.0, -0.8) |
| Wave 7 (1998-2001) | 3.4 (3.2, 3.7) | 1.1 (1.0, 1.2) | 3.4 (3.2, 3.6) | -1.0 (-1.1, -1.0) |
| Wave 8 (2005-2008) | 2.9 (2.6, 3.2) | 0.8 (0.7, 0.9) | 5.2 (5.0, 5.4) | -1.1 (-1.2, -1.0) |

Note: All analyses are adjusted for age, sex, education, and census tract poverty

Table S3. Main Results for Multilevel Models Examining Food Environments over Time based on the Workplace Address

| Distance from Closest | | | | |
| --- | --- | --- | --- | --- |
|  | Full Service Restaurant | Bakeries and Coffee Shops | Convenience Store | Grocery Store |
|  | Change in Meters (95% CI) | Change in Meters (95% CI) | Change in Meters (95% CI) | Change in Meters (95% CI) |
| Wave 1 (1971-1975) | Ref | Ref | Ref | Ref |
| Wave 2 (1979-1983) | -34.8 (-54.8, -14.8) | -59.9 (-99.9, -20.0) | -218.7 (-248.8, -188.7) | 125.5 (81.4, 169.6) |
| Wave 3 (1983-1987) | -37.0 (-58.9, -15.1) | -190.2 (-231.1, -149.2) | -340.9 (-372.3, -309.5) | 186.1 (141.3, 230.9) |
| Wave 4 (1987-1991) | -111.1 (-136.7, -85.5) | -222.7 (-268.5, -177.0) | -364.0 (-399.5, -328.4) | 588.5 (538.9, 638.2) |
| Wave 5 (1991-1995) | -142.3 (-172.8, -111.9) | -293.0 (-348.2, -237.9) | -380.0 (-422.0, -337.9) | 511.9 (451.6, 572.2) |
| Wave 6 (1995-1998) | -123.8 (-157.0, -90.6) | -458.3 (-515.5, -401.0) | -377.1 (-421.6, -332.6) | 298.1 (236.4, 359.7) |
| Wave 7 (1998-2001) | -104.7 (-145.7, -63.7) | -356.9 (-426.9, -286.9) | -386.4 (-440.1, -332.7) | 235.1 (159.2, 310.9) |
| Wave 8 (2005-2008) | -31.6 (-83.5, 20.2) | -401.9 (-490.9, -312.9) | -658.2 (-726.1, -590.4) | 257.8 (161.6, 354.0) |
| Counts within 1500m Buffer | | | | |
|  | Full Service Restaurants | Bakeries and Coffee Shops | Convenience Stores | Grocery Stores |
|  | Change in Count | Change in Count | Change in Count | Change in Count |
|  | (95% CI) | (95% CI) | (95% CI) | (95% CI) |
| Wave 1 (1971-1975) | Ref | Ref | Ref | Ref |
| Wave 2 (1979-1983) | 0.8 (0.6, 1.0) | -0.5 (-0.6, -0.4) | 0.5 (0.4, 0.6) | -0.8 (-0.9, -0.7) |
| Wave 3 (1983-1987) | 1.3 (1.1, 1.6) | -0.5 (-0.6, -0.4) | 1.2 (1.1, 1.3) | -0.6 (-0.7, -0.5) |
| Wave 4 (1987-1991) | 2.7 (2.4, 3.0) | -0.1 (-0.2, 0.0) | 1.1 (1.0, 1.2) | -0.9 (-1.0, -0.8) |
| Wave 5 (1991-1995) | 4.3 (3.9, 4.6) | 0.6 (0.5, 0.8) | 1.9 (1.8, 2.1) | -1.1 (-1.2, -1.0) |
| Wave 6 (1995-1998) | 4.2 (3.8, 4.6) | 1.6 (1.5, 1.8) | 2.9 (2.8, 3.1) | -1.4 (-1.5, -1.2) |
| Wave 7 (1998-2001) | 3.5 (3.1, 4.0) | 1.4 (1.2, 1.6) | 3.0 (2.8, 3.2) | -1.2 (-1.4, -1.1) |
| Wave 8 (2005-2008) | 3.3 (2.7, 3.9) | 1.1 (0.9, 1.3) | 5.1 (4.8, 5.3) | -1.1 (-1.3, -0.8) |

Note: All analyses are adjusted for age, sex, education, and census tract poverty

Table S4. Main Results for Multilevel Models Examining Food Environments over Time based on the Commute from Work to Home

| Counts within 60m Buffer | | | | |
| --- | --- | --- | --- | --- |
|  | Full Service Restaurants | Bakeries and Coffee Shops | Convenience Stores | Grocery Stores |
|  | Change in Count | Change in Count | Change in Count | Change in Count |
|  | (95% CI) | (95% CI) | (95% CI) | (95% CI) |
| Wave 1 (1971-1975) | Ref | Ref | Ref | Ref |
| Wave 2 (1979-1983) | 0.3 (0.0, 0.6) | -0.1 (-0.2, 0.0) | 0.3 (0.2, 0.4) | -0.3 (-0.4, -0.2) |
| Wave 3 (1983-1987) | 0.7 (0.4, 1.1) | 0.1 (-0.1, 0.2) | 0.9 (0.8, 1.0) | -0.2 (-0.3, 0.0) |
| Wave 4 (1987-1991) | 1.7 (1.3, 2.0) | 0.4 (0.3, 0.6) | 1.1 (0.9, 1.3) | 0.1 (0.0, 0.2) |
| Wave 5 (1991-1995) | 2.4 (2.0, 2.8) | 0.7 (0.5, 0.8) | 1.6 (1.4, 1.8) | 0.3 (0.1, 0.4) |
| Wave 6 (1995-1998) | 2.5 (2.1, 3.0) | 1.2 (1.0, 1.4) | 1.9 (1.6, 2.1) | 0.0 (-0.1, 0.2) |
| Wave 7 (1998-2001) | 2.7 (2.2, 3.3) | 1.1 (0.9, 1.3) | 2.0 (1.7, 2.3) | -0.3 (-0.5, -0.1) |
| Wave 8 (2005-2008) | 1.9 (1.1, 2.6) | 0.5 (0.2, 0.8) | 3.2 (2.8, 3.6) | -0.4 (-0.6, -0.1) |

Note: All analyses are adjusted for age, sex, education, and census tract poverty
